# Effects of Palmitoylethanolamide (PEA) on Nociceptive, Musculoskeletal and Neuropathic Pain: Systematic Review and Meta-Analysis of Clinical Evidence

**DOI:** 10.3390/pharmaceutics14081672

**Published:** 2022-08-11

**Authors:** Damiana Scuteri, Francesca Guida, Serena Boccella, Enza Palazzo, Sabatino Maione, Juan Francisco Rodríguez-Landa, Lucia Martínez-Mota, Paolo Tonin, Giacinto Bagetta, Maria Tiziana Corasaniti

**Affiliations:** 1 Pharmacotechnology Documentation and Transfer Unit, Preclinical and Translational Pharmacology, Department of Pharmacy, Health and Nutritional Sciences, University of Calabria, 87036 Rende, Italy; 2 Regional Center for Serious Brain Injuries, S. Anna Institute, 88900 Crotone, Italy; 3 Department of Experimental Medicine, Pharmacology Division, University of Campania “L. Vanvitelli”, 80138 Naples, Italy; 4 Endocannabinoid Research Group, Institute of Biomolecular Chemistry, CNR, 80078 Pozzuoli, Italy; 5 IRCSS, Neuromed, 86077 Pozzilli, Italy; 6 Laboratorio de Neurofarmacología, Instituto de Neuroetología, Universidad Veracruzana, Xalapa 91190, Mexico; 7 Facultad de Química Farmacéutica Biológica, Universidad Veracruzana, Xalapa 91001, Mexico; 8 Dirección de Investigaciones en Neurociencias, Instituto Nacional de Psiquiatría Ramón de la Fuente Muñiz, Mexico City 03440, Mexico; 9 Department of Health Sciences, University “Magna Graecia” of Catanzaro, 88100 Catanzaro, Italy

**Keywords:** palmitoylethanolamide, PEA, nociceptive pain, neuropathic pain, clinical setting

## Abstract

Some 30–50% of the global population and almost 20% of the European population actually suffer from chronic pain, which presents a tremendous burden to society when this pain turns into a disability and hospitalization. Palmitoylethanolamide (PEA) has been demonstrated to improve pain in preclinical contexts, but an appraisal of clinical evidence is still lacking. The present study aimed at addressing the working hypothesis for the efficacy of PEA for nociceptive musculoskeletal and neuropathic pain in the clinical setting. The systematic search, selection and analysis were performed in agreement with the Preferred Reporting Items for Systematic Reviews and Meta-Analyses (PRISMA) 2020 recommendations. The primary outcome was pain reduction, as measured by a pain assessment scale. The secondary outcome was improvement in quality of life and/or of parameters of function. The results obtained for a total of 933 patients demonstrate the efficacy of PEA over the control (*p* < 0.00001), in particular in six studies apart from the two randomized, double-blind clinical trials included. However, the results are downgraded due to the high heterogeneity of the studies (I^2^ = 99%), and the funnel plot suggests publication bias. Efficacy in achieving a reduction in the need for rescue medications and improvement in functioning, neuropathic symptoms and quality of life are reported. Therefore, adequately powered randomized, double-blind clinical trials are needed to deepen the domains of efficacy of add-on therapy with PEA for chronic pain. PROSPERO registration: CRD42022314395.

## 1. Introduction

Chronic pain is one of the most common reasons for seeking clinical assistance [1], due to a reduced level of functioning and risk for addiction to painkillers with a tremendous impact on quality of life. In particular, functional disability increases due to the neuropathic components of chronic pain. Among the most activity-limiting syndromes is lower-back pain [2], a major problem throughout the world with a lifetime prevalence of about 39% [3]. Neuropathic pain, being caused by a lesion or disease of the somatosensory system [4], can be due to spinal surgery procedures [5] or injury [6] and also stroke [7], but also to neuropathies caused by comorbidities reaching up to 60% in prevalence, such as diabetes [8] and shingles due to herpes zoster infection, as supported by several pathogenetic hypotheses [9]. Moreover, it is now widely known that patients with common rheumatic conditions present neuropathic features [10,11,12], in spite of the nociceptive and inflammatory nature of this type of pain [13]. All these painful conditions increase with the aging of the population, making chronic musculoskeletal pain the leading cause of disability among the elderly [14,15]. In this fragile population, the issue of pain is even more difficult to deal with due to the lack of information on appropriate use of analgesics, in terms of tolerance and addiction (for opioids as well as gabapentinoids) [16,17,18,19], dosage and type, physiological differences and variability in pain processing [20], response to drugs [21] and polypharmacy [22]. This lack of information is due to the practice of excluding older patients from clinical trials [23], mainly for antimigraine treatments [24,25,26]. Incidentally, the elderly most often are affected by dementia, causing underdiagnosis [27] and undertreatment of pain in the least investigated community contexts [28], with remarkable burden in terms of agitation [29]. Since chronic musculoskeletal and neuropathic pain mainly affects older patients (instead of migraines, which affect a high percentage of young people) and is often resistant to treatment, the use of natural products with proven analgesic properties is the safest option, although rigorous, reliable clinic research is still needed to confirm this [30]. Preclinic research from our group built the rationale for the clinical translation of the essential oil of bergamot [28,31,32,33,34,35]. Among the different compounds investigated for their analgesic efficacy, palmitoylethanolamide (PEA) is an endogenous fatty acid amide with antioxidant activity [36] that is believed to be produced in the bilayer in response to injury [37], exerting pleiotropic actions, including: indirect endocannabinoid modulation [38,39] through inhibition of fatty acid amide hydrolase (FAAH) [40]; activity of the nuclear receptor peroxisome proliferator-activated receptor-α (PPAR-α) [41]; and activation of the transient receptor potential channel of the vanilloid type 1 (TRPV1) [42]. The preclinic analgesic properties of PEA and its derivatives, micronized and ultramicronized and combined with other compounds, demonstrate its critical role in the modulation of pain-related behaviors by acting on glutamatergic neurotransmission, as shown in pain-related studies and mild traumatic brain injury animal models [43,44]. A systematic review and meta-analysis of studies that assessed the antinociceptive efficacy of cannabinoids, cannabis-based medicines and endocannabinoid system modulators for pain-associated behavioral outcomes in animal models of pain demonstrated the efficacy of these compounds for attenuating pain-associated behaviors, but revealed an unclear risk of bias, highlighting the need for adequate research and reporting methodologies [45]. A meta-analysis of randomized trials demonstrated pain reduction in a very small number of studies [46], and a scoping review protocol is under investigation [47]. The aim of the present systematic review and meta-analysis was to appraise, for the first time, the clinical evidence in favor of the efficacy of PEA for nociceptive, musculoskeletal and neuropathic pain in randomized and nonrandomized studies, with a blinded or open-label and observational design following the most updated Preferred Reporting Items for Systematic Reviews and Meta-Analyses (PRISMA) 2020 recommendations [48].

## 2. Materials and Methods

### 2.1. Objectives and Protocol

The PRISMA 2020 recommendations [48,49,50] were followed to answer to the PICOS (participants/population, interventions, comparisons, outcomes and study design) question. In particular, the intervention consisted of PEA administered alone or in combination via any route. Eligible studies were those that compared the effect of the intervention with a placebo/no treatment or an active control, i.e., medications found to be effective and approved for pain treatment. Eligible studies included prospective and retrospective clinical studies. The primary efficacy outcome consisted of pain reduction as measured with a pain assessment scale. The secondary outcome was improvement in quality of life and/or of parameters of function. The protocol of the systematic review and meta-analysis was established prior to the literature search, and it is registered in the National Institute for Health Research (NIHR) international prospective register of systematic reviews PROSPERO (CRD42022314395). The search, extraction and selection of the retrieved studies, as well as analysis of results, were performed in agreement with the most recently updated PRISMA 2020 recommendations [48]. Two independent review committee members screened titles and abstracts and, subsequently, the full text of the retrieved studies, according to the a priori established inclusion and exclusion criteria. The reference list of relevant papers was inspected to prevent potentially missing additional studies in the database search. We planned to solve any disagreement by consensus or by consulting a third team member.

### 2.2. Eligibility Criteria

The analysis included patients of any age, ethnicity and gender suffering from nociceptive musculoskeletal or neuropathic pain. Studies not eligible for the analysis were: in vitro and in vivo animal studies; narrative or systematic reviews and meta-analysis; abstracts and congress communications; proceedings; editorials and book chapters; studies not available in a full-text format; and studies not published in English. No restrictions concerned with study duration, follow-up or publication date were applied. The inclusion and exclusion criteria are reported in Table 1.

### 2.3. Information Sources

The most relevant databases for medical, scientific literature were screened for peer-reviewed studies published in databases from their inception to the present: PubMed/MEDLINE, Scopus, Web of Science (WOS) and Cochrane Library databases (Cochrane Central Register of Controlled Trials-CENTRAL). Screening for additional unpublished studies was performed on the ClinicalTrials.gov registry. The process of database screening was conducted by two independent members of the review committee for records matching the search terms up to the date of the last search on 22 May 2022.

### 2.4. Search Strategy

The following medical and subject headings (MeSH) terms were used in combination: “Palmitoylethanolamide”; “co-ultraPEALut”; “Pain”; “Acute Pain”; “Musculoskeletal Pain”; “Chronic Pain”; “Nociceptive Pain”; and “Neuropathic Pain”. The aim was to carry out a high-sensitivity/recall search strategy maintaining precision [51]. The search on the ClinicalTrials.gov registry used the string “Palmitoylethanolamide AND Pain”. No validated search filters for study design were found [51]. Based on the evidence-based guideline for Peer Review of Electronic Search Strategies (PRESS) for systematic reviews (SRs) [51,52], an author different (reviewer) from the two searching the databases independently (requestors) peer-reviewed the search strategy, ensuring: (1) the accuracy of lines and spelling of search strings; (2) the appropriateness of the search regarding its coverage of all the most relevant aspects; and (3) correctness of interpretation and answer to the participants, intervention, comparator, outcome and study design (PICOS) questions.

### 2.5. Study Selection

The risk of missing relevant records was minimized through independent eligibility assessment of the studies by two authors. Duplicate records were removed using reference manager software (EndNote X7, Clarivate, London, UK). Subsequently, the title and abstract, first, and the full text, later, were screened. The reference list of the retrieved records was checked to extend and refine the search. The overall consensus among all the authors, without the occurrence of relevant conflicts, which we planned to solve through the Delphi method [53], was reported.

### 2.6. Data Synthesis, Risk of Bias Assessment and Critical Appraisal

The synthesis of the results followed the Cochrane Consumers and Communication Review Group guidelines [54], considering: the report (author and year); the study design and sample size; the participants, based on type of pain; the research design with sampling, treatment assignment, any randomization, allocation and concealment mechanisms; and the intervention type, timing and dose and study duration. The risk of bias (RoB) in the results of the individual studies and in the studies’ synthesis and the quality/certainty [55] of the body of evidence, according to the PRISMA 2020 statement [56], was evaluated independently by two members of the review committee. The revised Cochrane risk of bias tool, RoB2, was used for randomized clinical trials (RCTs) [57], and the Risk Of Bias In Non-randomized Studies of Interventions (ROBINS-I) tool [58] was used for studies not using an RCT design. Any discrepancies in judgement of risk of bias were resolved through a discussion between the two review authors to reach a consensus, consulting a third author if necessary. The visualization of the risk of bias assessment was produced with the Cochrane robvis visualization tool [59].

### 2.7. Statistical Analysis and Effect Measures

Standardized mean differences (SMDs) and inverse variance were calculated for continuous variables through the Cochrane Review Manager 5.3 (RevMan5.3; Copenhagen: The Nordic Cochrane Center, The Cochrane Collaboration). No sensitivity analysis (i.e., restricting the primary analysis to low-risk-of-bias studies) or following subgroup analysis or meta-regression based on stratification of the studies according to the judgement of the risk of bias was performed because of the small number of studies meeting the inclusion criteria. The random-effect model [60] and the Higgins I^2^ value [61] were used to evaluate the heterogeneity of the studies. The publication bias was assessed through Egger’s linear regression test [62] for funnel plot asymmetry [63], adjusted through the “trim and fill” method [64].

## 3. Results

### 3.1. Extraction of the Studies

**Figure 1:**
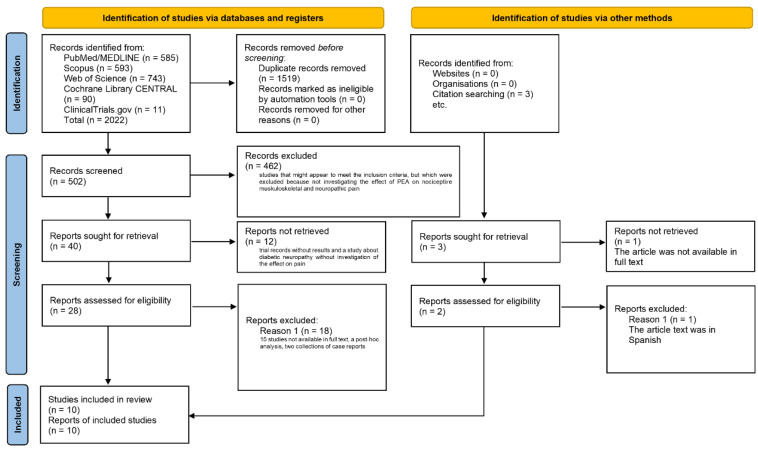
Process of search, selection and identification of studies according to the Preferred Reporting Items for Systematic Reviews and Meta-Analyses (PRISMA) 2020 flow diagram for new systematic reviews, which included searches of databases, registers and other sources.

The search of databases retrieved 2022 results: 585 records from PubMed/MEDLINE; 593 records from Scopus; 743 records from WOS; 90 records from Cochrane Library (CENTRAL); and 11 records from ClinicalTrials.gov. Three studies were identified using other methods, i.e., reference list screening, including: the study by Gatti et al. [65], which met the inclusion criteria, and thus was included; the study of Desio [66], which had to be excluded because of the lack of a full text; and the article of Canteri and collaborators [67], which could not be included because it was in Spanish. After duplicate removal (1520 duplicated studies), there were 502 records left. Title and abstract screening led to the elimination of the studies not meeting the inclusion criteria because they used a different design (studies not of a clinical nature, reviews, chapters and congress abstracts) or due to the intervention used (studies that might appear to meet the inclusion criteria, but were excluded because they did not investigate the effect of PEA on nociceptive musculoskeletal and neuropathic pain) and of the following records on: endometriotic/pelvic pain (NCT02372903, NCT04091789, [68,69,70,71,72,73,74,75,76,77,78,79]); mouth pain [80,81,82]; and irritable bowel syndrome [83,84]. Therefore, 40 full-text studies were left to assess. Of these, 15 records were not available [85,86,87,88,89,90,91,92,93,94,95,96,97,98]. The Ms by Pieralice and colleagues [99] was excluded because it investigated the effect of PEA on diabetic neuropathy, but not on pain. Moreover, trial records without results were excluded, including: NCT01851499, NCT01851499, NCT01491191, ACTRN12619000418178, ACTRN12621000039886, ACTRN12620001302943, NCT05317676, ACTRN12615000149561, CTRI/2021/08/036091, NCT04662827 and ACTRN12621000228886. The study by Cruccu and coworkers [100] was excluded because it was a post hoc analysis of an excluded record not available in full text, and the studies by Hesselink and collaborators [101,102] were not included because they comprised a collection of seven case reports. Finally, 10 studies were included in the analysis, and 8 of them presenting comparable measures of the primary outcome (pain intensity reduction) were subjected to the meta-analysis. The process of selection of studies is illustrated in Figure 1.

### 3.2. Synthesis of the Studies

Among the studies meeting all the inclusion criteria, two were double-blinded RCTs [103,104], and only one of them [103] was based on a sample power calculation. The other eight studies [65,95,105,106,107,108,109,110] were observational and open-label studies. A total of 1116 patients treated with PEA and its derivatives included in the data synthesis suffered from all conditions associated with resistant chronic pain. The double-blind RCT by Andresen et al. reports the absence of a statistically significant effect of ultramicronized PEA (PEA-um) on the primary outcome of reduction in pain intensity as assessed by a numeric rating scale (NRS; PEA-um 6.3 ± 1.7 and 0.4 ± 1.4 from baseline; placebo 5.5 ± 1.8 and 0.7 ± 1.4 from baseline). However, a significant reduction in the use of rescue medication is highlighted. Five patients reported serious adverse events (urinary tract infection, paralytic ileus, cholecystolithiasis, erysipelas causing hospitalization, fungus infection, blurred vision). An increase in self-reported intensity of spasticity was found. In the study by Bonetti and colleagues, the combined treatment of oxygen–ozone therapy with oral alpha-lipoic acid (800 mg/day), PEA (600 mg/day) and myrrh (200 mg) afforded complete remission of pain in 70.3% of the patients. Oral PEA-um treatment was investigated in the study by Cocito and collaborators, which found a significant reduction in the visual analogue scale (VAS) mean score after 40 days of treatment (5.80 ± 2.04; *p* < 0.001), together with significant improvement in the neuropathic pain symptom inventory (NPSI) total score. The double-blind RCTs by Faig-Martì and Martinez-Catassus reported no significant differences in any outcomes, with a VAS score of 3.76 ± 3.19 for PEA vs. 3.25 ± 3.18 for the control after 60 days of treatment. The study by Gatti and coworkers showed a significant decrease in the NRS score from 6.4 ± 1.4 to 2.5 ± 1.3, without treatment-related adverse events. One month of PEA treatment 2–8 months after the surgical procedure was found to reduce the VAS score (4.3 ± 0.11 vs. 5.7 ± 0.12) in the study of Paladini et al. The study of Parisi et al. involved patients with different forms of neuropathic pain (sciatic pain, carpal tunnel syndrome and peripheral neuropathy of the lower limbs), demonstrating the efficacy of a fixed combination of PEA (600 mg) + Acetyl-L-Carnitine (500 mg) (Kalanit^®^) vs. standard therapy on the scores of the VAS, Low Back Pain Impact Questionnaire (LBP-IQ), cochin hand functional disability (CHFD) scale and Neuropathic Pain Questionnaire (NPQ). The study by Passavanti and collaborators showed the effects of PEA-um as an add-on to tapentadol therapy for 6 months, with paracetamol (1000 mg) used as a rescue drug, showing a significant reduction in the VAS score from 7.4 ± 0.08 to 4.5 ± 0.09 and a reduction in the score of the neuropathic component (Doleur Neuropatique 4, DN4) and the degree of disability (Oswestry Disability Questionnaire, ODQ). In the study by Scaturro and colleagues, 600 mg of PEA-um given twice a day in combination with a daily functional rehabilitation session induced a significant decrease in the score on the NRS from 6.3 ± 0.1 at baseline to 3.7 ± 0.09 and 2 ± 0.09 at 30 and 60 days, respectively, accompanied by a significant improvement in quality of life and the mental component. In the study by Schifilliti et al., treatment with micronized palmitoylethanolamide (300 mg twice daily) for 60 days significantly reduced the pain symptoms characteristic of diabetic neuropathy (Michigan Neuropathy Screening Instrument (MNSI); Total Symptom Score (TSS); diabetic neuropathic pain symptoms; NPSI) after 30 days with no serious adverse events.

### 3.3. Risk of Bias

**Figure 2:**
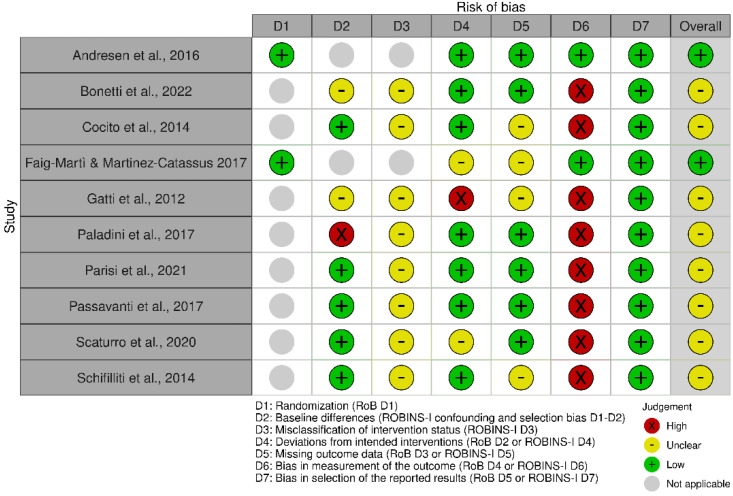
Robvis traffic light plot for the risk of bias of the studies included in the analysis. The studies’ RoB was assessed, according to their study design, with the revised Cochrane risk of bias tool for randomized trials, RoB2, and the Risk Of Bias In Non-randomised Studies of Interventions tool (ROBINS-I) for the assessment of effectiveness or safety (benefit or harm) of an intervention from nonrandomized studies on the effects of interventions (NRSI). The items assessed for RoB were: randomization; deviations from intended interventions; missing outcome data; bias in measurement of the outcome; and bias in selection of the reported results. Due to the nonrandomized study design, the outcomes assessed by ROBINS-I include baseline differences and misclassification of intervention status instead of the randomization domain.

**Figure 3:**
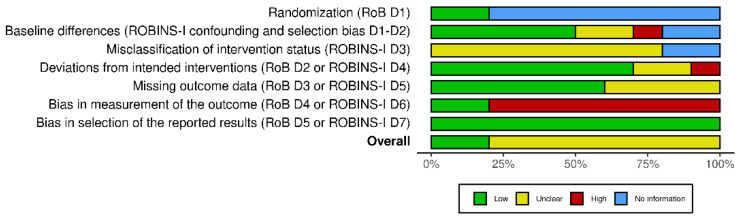
Robvis summary plot for the risk of bias (RoB) of the studies included in the analysis. The studies’ RoB was assessed, according to their study design, with the revised Cochrane risk of bias tool for randomized trials, RoB2, and the Risk Of Bias In Non-randomised Studies of Interventions tool (ROBINS-I) for the assessment of effectiveness or safety (benefit or harm) of an intervention from nonrandomized studies on the effects of interventions (NRSI). The items assessed by RoB are: randomization; deviations from intended interventions; missing outcome data; bias in measurement of the outcome; and bias in selection of the reported results. Due to the nonrandomized study design, the outcomes assessed by ROBINS-I include baseline differences and misclassification of intervention status instead of the randomization domain.

For the randomized clinical trials included in the analysis, risk of bias was assessed considering study limitations, including the lack of allocation concealment, lack of blinding, selective outcome reporting bias and inadequate sample size or lack of sample size calculation, according to the revised Cochrane risk of bias tool for randomized trials, RoB2 [57]. On the contrary, the observational and open-label studies’ risk of bias was evaluated with the ROBINS-I tool [58] for the assessment of effectiveness or safety (benefit or harm) of an intervention from nonrandomized studies on the effects of interventions (NRSI). It consisted of the assessment of the following seven domains: confounding and selection of participants (pre-intervention bias, differing from randomized trial bias assessment); classification of the interventions (at intervention bias, differing from randomized trial bias assessment); deviations from intended interventions, missing data, measurement of outcomes and selection of the reported result (post-intervention bias, not differing from randomized trial bias assessment). Due to their design, the only studies to achieve an overall low risk of bias rating were the two RCTs by Andresen et al. and Faig-Martì and Martinez-Catassus (Figure 2, traffic light plot of risk of bias assessment), showing the lack of randomization and blinding in the other studies (Figure 3, summary plot of risk of bias assessment). In the studies in which data about missing outcomes or deviation from intervention are not quoted, they are considered not to have occurred. In the study by Bonetti et al., baseline statistical differences are not reported; therefore, the risk of bias is unclear, as well as the scale used to measure pain. During the study by Cocito and coworkers, three subjects dropped out for reasons not related to PEA-um administration (one patient underwent surgery for cholecystitis, one patient had a vertebral fracture, requiring hospitalization, and one patient had an intestinal virus). In the study by Gatti et al., 46 patients dropped out. In any cases of drop-out, some concerns in terms of missing outcome data bias are reported in the analysis. Moreover, the need for differences in the treatment could represent a bias found in the study of Gatti and collaborators and of Scaturro and colleagues, in which “conventional therapies were adjusted according to dose, optimized and administered to each patient at a fixed dose throughout the entire observational period”. These two studies had the greatest sample size (n = 610 and n = 120, respectively). The requirement for pre-treatment with tapentadol and pregabalin occurring in the study by Paladini et al. could induce confounding bias. Deidentification of data was not reported by any of the non-RCTs that, thus, present bias in measurement outcomes. Differential misclassification was present in all the non-RCT studies, since none reported the absence of knowledge of the outcomes at the moment of the allocation to the intervention group.

### 3.4. Meta-Analysis

**Figure 4:**
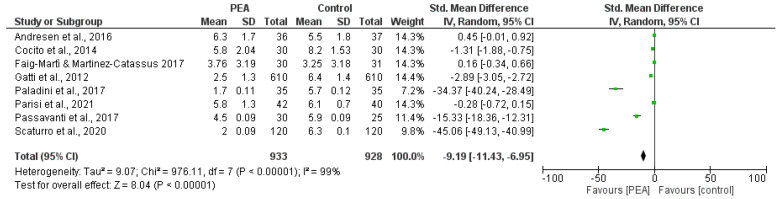
Summary of mean and standard deviation (SD) of the reduction in pain intensity scores assessed through the numeric rating scale (NRS)/visual analog scale (VAS) of the eight studies eligible for quantitative analysis. The forest plot of the meta-analysis of the records demonstrates efficacy of the intervention over the comparator, but in the presence of high heterogeneity of the studies (I^2^ = 99%).

**Figure 5:**
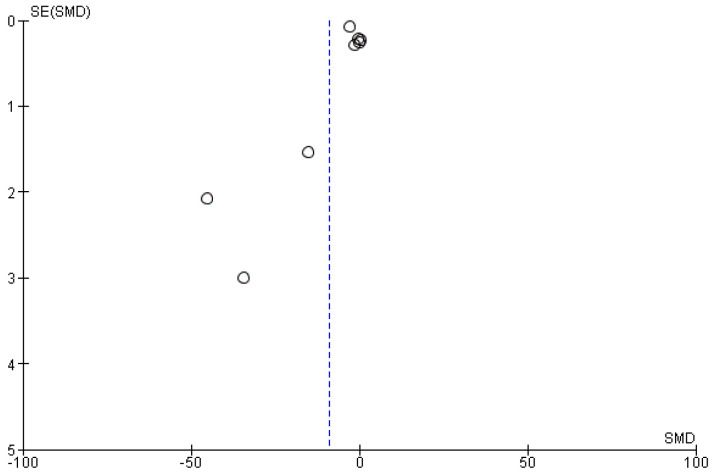
Funnel plot. Publication bias of the studies is highlighted by asymmetry of the graph.

The most homogeneous outcome across the studies included to conduct the meta-analysis is represented by reduction of NRS/VAS scores of pain intensity, in spite of great heterogeneity in intervention types and schedules. The highest follow-up measure was chosen. Therefore, the studies included in the meta-analysis were the following 8 out of the 10 total studies, not including the studies by Bonetti et al. and Schifilliti et al. The results obtained for a total of 933 patients demonstrate the efficacy of PEA over controls, in particular in six cases (the two RCTs by Andresen et al. and Faig-Martì and Martinez-Catassus do not report the clinic analgesic efficacy of PEA), three of which exhibited high CIs, in a statistically significant manner (*p* < 0.00001). However, the results are downgraded due to the high heterogeneity of the studies (I^2^ = 99%) (Figure 4). The funnel plot suggests publication bias (Figure 5).

The eight articles eligible for meta-analysis were grouped and analyzed based on the Cochrane Consumers and Communication Review Group guidelines. The main characteristics of the studies investigated are summarized in Table 2, and all the features, including study design, participants, exposure and assessment, the risk of bias assessment and the meta-analysis, are reported in the Graphical Overview for Evidence Reviews (GOfER) diagram [111]. The GOfER is provided as Supplementary Material illustrating: the report (author and year); the study design and sample size; the participants’ baseline characteristics and the inclusion criteria; the research design with treatment assignment, allocation and concealment mechanisms and length of follow up; and the intervention type, timing, dose and the duration of the study.

## 4. Discussion

The systematic search of databases according to PRISMA 2020 recommendations retrieved 2022 results. Of these, ten records met the inclusion criteria and only eight shared comparable primary outcomes of pain intensity reduction and were thus included in the meta-analysis. The secondary outcome measures were not comparable among studies, thus making the meta-analysis not feasible [112]. Only two of these studies with the full text available were RCTs, while the others had a nonrandomized and blinded, open-label or observational design. The lack of adequately powered RCTs emerged; in fact, the nonrandomized and blinded, open-label or observational study design was a limitation of all the trials eligible apart from those by Andresen et al. and Faig-Martì and Martinez-Catassus, which present randomization and blinding as strengths. Other limitations linked to non-RCT study designs occurred in all the studies apart from the studies by Andresen et al. and Faig-Martì and Martinez-Catassus, which included a lack of deidentification of data and high risk of misclassification of the outcome measures. Moreover, the strengths of the study of Gatti and collaborators and the study of Scaturro and colleagues rely on the greatest sample sizes enrolled. The greatest strength of all the studies was the absence of reported missing outcome data due to drop-out. However, concerns regarding drop-out in terms of missing outcome data bias represent limitations of the trials by Faig-Martì and Martinez-Catassus, Gatti and collaborators and Scaturro and colleagues. Furthermore, during the study by Cocito and coworkers, three subjects dropped out for reasons not related to PEA-um administration. As previously demonstrated for the efficacy of nutraceuticals in treating glaucoma [113], in the research field of dietary supplements, the number of RCTs is poor. The results obtained for a total of 933 patients demonstrate the efficacy of PEA over controls (*p* < 0.00001), in particular in the six nonrandomized studies included. However, this clinical analgesic efficacy is not generalizable because of the high heterogeneity of the studies (I^2^ = 99%) and the funnel plot suggests publication bias. This is in agreement with the finding of lots of studies not available as a full text. Efficacy in achieving a reduction in the need for rescue medication and improvement in functioning, neuropathic symptoms and quality of life is reported. In fact, although reporting the absence of a statistically significant effect of PEA-um on pain intensity reduction, the double-blind RCT by Andresen et al. points at a significant reduction in the use of rescue medications, represented by paracetamol (Δ mean:−2.2, 95% CI:−4.0 to−0.3, *p* = 0.02) [103]. Therefore, according to the authors, the main effect of PEA could be due to anti-inflammatory and neuroprotective properties elicited in animal models [114]. In fact, PEA is endowed with several actions, including: ATP-sensitive K ± channels [115]; downmodulation of mast-cell activation [116] in inflammation; and inhibition of nuclear factor kB (NF-kB) signaling in the dorsal horn [117]. The reduction in paracetamol needed as rescue medication is reported also in the study by Passavanti and collaborators [95]. In the overall study evaluation PEA is well-tolerated. The study of Parisi et al. [110] demonstrated the efficacy of Kalanit^®^ for CHFD as well, in agreement with the study by Passavanti et al. [95] showing its efficacy in achieving a decrease in the ODQ score and with the study by Scaturro and colleagues [108] reporting significant improvement in quality of life and the mental component. The need for further research in the field and a head-to-head comparison of different PEA formulations is supported by the study of Gabrielsson et al. [118], showing through access to raw data of trials, open-label and case-control studies, that the quality of key studies is poor. The most adequately powered study not included in this analysis as it is not available as a full text is the study by Guida et al., which enrolled 636 patients with lumbosciatic algias and proved the efficacy of PEA [100,119]. A previous meta-analysis dating back to 2017 [46] had already highlighted the small number of trials assessing pain due to widely differing conditions, thus pointing at the various study designs for obtaining data in this research field. These are great limitations that do not allow for definite conclusions to be drawn about the efficacy of PEA for the reduction in pain intensity and the need for rescue medications. The present meta-analysis demonstrates the statistically significant (*p* < 0.00001) efficacy of PEA over controls for a total of 933 patients, although the two RCTs by Andresen et al. and Faig-Martì and Martinez-Catassus did not show the efficacy of PEA. As previously described, the study limitations include the high heterogeneity of the trials eligible (I^2^ = 99%), the risk of bias due to non-RCT study design and the highlighted publication bias. Therefore, the findings on the efficacy of PEA for the reduction in pain intensity and the need for rescue medications require confirmation through rigorous RCTs on musculoskeletal and neuropathic pain.

## 5. Conclusions

Chronic pain is a widespread condition that remarkably reduces the patients’ quality of life. Patients do not find sufficient relief from current therapies, which induce adverse reactions that can be serious in older fragile patients. Adequately powered randomized, double-blind clinical trials are needed to deepen the domains of efficacy of add-on therapy with PEA for chronic pain. In particular, the design of future studies needs to be homogeneous to allow for comparison, network and pooled data analyses and following the quality standard for evidence production. One of the main outcomes that deserves particular attention in the clinical investigation of the properties of PEA is the reduction in the need for rescue medications; in fact, it could reduce the dosage of painkillers, minimizing their side effects, which, in almost half of cases, can be avoided and are known to lead to geriatric unit admission [120]. This point is of the utmost importance for the elderly subjected to polypharmacy due to comorbidities [22] and is linked to inappropriate prescriptions [121]. In fact, according to the Italian Silver Network Home Care project, some 49% patients are affected by daily pain, being treated with World Health Organization (WHO) level I analgesics in the 25% of case only [122]. The latter also concerns the poorly investigated herbal-drug interactions occurring in the older patients [123]. To obtain the necessary high-quality evidence for the efficacy of PEA for the reduction in the need for rescue medications, an adequate sample size, based on calculations according to the current literature and including older patients suffering from musculoskeletal, neuropathic and mixed origin pain, is required to conduct an RCT assessing PEA add-on therapy efficacy and safety and the concurrent decrease in dosage of analgesics such as coxibs, opioids and gabapentinoids. Additionally, neuropathic symptoms and improvement in functioning and quality of life deserve appropriate assessment.

## Figures and Tables

**Table 1:** Inclusion and exclusion criteria for the extraction and selection of results from database search.

Inclusion Criteria	Exclusion Criteria
Patients of any age, gender and ethnicity suffering from pain;	Animal studies;
	Publications reporting on in vitro studies;
	Case reports;
	Narrative reviews;
	Systematic reviews and meta-analysis;
	Abstracts and congress communications, proceedings, editorials and book chapters;
	Editorials;
No restrictions regarding study duration or follow-up and publication date.	Studies not available in a full-text format or not published in English.

**Table 2:** Extract of the Graphical Overview for Evidence Reviews (GOfER) diagram of the systematic review and meta-analysis. The studies eligible for inclusion in the systematic review were analyzed based on their design, exposure to intervention and comparator and results from assessment of the outcome measures.

Study Report	Study Design	Intervention	Control	Results
Andresen et al., 2016	Randomized, double-blind, placebo-controlled, parallel multicenter trial NCT01851499	Sublingual ultramicronized PEA (PEA-um) 600 mg (Normast^®^), twice daily with approximately 12 h between doses for 12 weeks, as add-on therapy. n = 36	Identical placebo n = 37	No statistically significant difference in primary outcome (PEA 6.3 ± 1.7 and 0.4 ± 1.4 from baseline, placebo 5.5 ± 1.8 and 0.7 ± 1.4 from baseline); significant reduction in the use of rescue medication; increase in self-reported intensity of spasticity; no statistically significant differences for any of the other secondary outcomes; 5 patients reported serious adverse events; urinary tract infection, paralytic ileus, cholecystolithiasis, erysipelas causing hospitalization, fungus infection, blurred vision
Bonetti et al., 2022	Observational	Combined treatment of oxygen–ozone therapy and oral treatment with alpha-lipoic acid (ALA, 800 mg/day) + palmitoylethanolamide (PEA, 600 mg/day) and myrrh (200 mg). n = 153. Three treatments with oxygen–ozone therapy over 30 days with period of 9 ± 2 days between the first and second therapeutic session and 18 ± 2 between the second and third	Oxygen–ozone treatment alone. n = 165. Three treatments with oxygen–ozone therapy over 30 days with period of 9 ± 2 days between the first and second therapeutic session and 18 ± 2 between the second and third	116/165 patients in Group A had a complete remission of pain (70.3%), while 21 (12.7%) and 28 (17.0%) had no benefit from the treatment, reporting a partial remission of painful symptoms, while in Group B, 119/153 (77.8%) had a complete remission of pain, 13 (8.5%) considered the outcome of the treatment sufficient and 21 (13.7%) considered it to be insufficient
Cocito et al., 2014	Open-label study	Oral PEA-um treatment was initiated at the doses of 1200 mg/die in sachet formulation for the first 10 days and 1200 mg/die in tablet formulation between the 10th and 40th days. The dosages of all other therapies were maintained during the entire duration of the study. n = 30	-	Significant decrease in the VAS mean score at the first evaluation (T1; 8.20 ± 1.53 vs. 6.4 ± 1.83, *p* < 0.002), even more evident at the T2 evaluation (5.80 ± 2.04; *p* < 0.001). Significant improvement in the NPSI total score, from 5.2 ± 1.5 to the T2 (40 days) values of 3.8 ± 2.1 (*p*: 0.025), and a similar trend was seen for the EQ-5D mean score, from the T0 value of −0.30 ± 0.65 to the T2 value of 0.50 ± 0.34 (*p* < 0.001)
Faig-Martì and Martinez-Catassus 2017	Prospective, double-blinded, randomized study	300 mg of PEA twice a day over 60 days. n = 30	Placebo with exactly the same appearance twice a day for the same period. n = 31	No significant differences in any outcomes. VAS 3.76 ± 3.19 (PEA) vs. 3.25 ± 3.18 (Control)
Gatti et al., 2012	Observational study	PEA (600 mg) was administered twice daily for 3 weeks followed by single daily dosing for 4 weeks, in addition to standard analgesic therapies or as single therapy. n = 610	-	NRS significant decrease from 6.4 ± 1.4 to 2.5 ± 1.3. No treatment-related adverse events or serious adverse events
Paladini et al., 2017	Observational study	Tapentadol and pregabalin at variable doses, for three months in this study. One month after the start of standard treatment, um-PEA (Normast, Epitech Group SpA, Saccolongo, Italia) was added at 1200 mg/day (two 600 mg tablets daily) for one month followed by 600 mg/day for the next month. n = 35	-	VAS (2–8 months after surgery) 5.7 ± 0.12 vs. VAS 4.3 ± 0.11 after 1 month of treatment (and 2.7 ± 0.09 after two and 1.7 ± 0.11 after 3 months of treatment) (for all measures, 𝑝 < 0.0001)
Parisi et al., 2021	Prospective study	Standard therapy + a fixed combination of PEA (600 mg) + Acetyl-L-Carnitine (500 mg) (Kalanit^®^) twice a day for 2 weeks and then once a day for 6 months. n = 42	Standard therapy. n = 40	Significant improvement in pain VAS: intervention 5.8 ± 1.3 vs. 7.1 ± 1.3 with respect to standard therapy 6.1 ± 0.7 vs. 6.8 ± 0.7. Significant improvement in LBP-IQ and CHFD scores.
Passavanti et al., 2017	Pilot, observational study	Prospective arm: PEA-um as add-on therapy to tapentadol for 6 months. Paracetamol (1000 mg) was habitually used as rescue drug in the case of exacerbation of pain. n = 30	Retrospective arm: tapentadol for 6 months. Paracetamol (1000 mg) was habitually used as rescue drug in case of exacerbation of pain. n = 25	VAS significant reduction from 7.4 ± 0.08 to 4.5 ± 0.09 in the prospective group vs. from 7.7 ± 0.10 to 5.9 ± 0.09 in the retrospective group. DN4 mean score reduction from 6.1 ± 0.14 to 3.2 ± 0.13 with PEA vs. from 6.1 ± 0.09 to 5.0 ± 0.04 in the retrospective group. Prospective group presented ODQ reduction from 56.9 ± 1.55 to 37.7 ± 2.38 vs. retrospective group going from 54.6 ± 2.20 to 44.6 ± 3.02. PEA significantly reduced the dosage of tapentadol and the use of paracetamol. No serious side effects
Scaturro et al., 2020	Observational Study	PEA-um 600 mg twice a day in combination with a daily functional rehabilitation session + a decontracting massage for 20 consecutive days, followed by 600 mg of umPEA once a day for 40 days in addition to standard therapy. n = 120	-	NRS decreased significantly from 6.3 ± 0.1 at baseline to 3.7 ± 0.09 and 2 ± 0.09 at 30 and 60 days, respectively. Significant improvement in quality of life and mental component
Schifilliti et al., 2014	Open-label study	Micronized palmitoylethanolamide (300 mg twice daily) for 60 days. n = 30	-	Significant reduction in the pain symptoms characteristic of diabetic neuropathy after only 30 days (MNSI, TSS, NPSI). No serious adverse events

## Data Availability

The original data presented in the study are included in the article.

## Supplementary Materials

The following supporting information can be downloaded at: https://www.mdpi.com/article/10.3390/pharmaceutics14081672/s1.

## Abbreviations

CHFD	Cochin hand functional disability
DN4	Doleur Neuropatique 4
SRs	Evidence-based guideline for Peer Review of Electronic Search Strategies (PRESS) for systematic reviews
FAAH	Fatty acid amide hydrolase
GOfER	Graphical Overview for Evidence Reviews diagram
PROSPERO	International prospective register of systematic reviews
LBP-IQ	Low Back Pain Impact Questionnaire
MeSH	Medical and subject headings
MNSI	Michigan Neuropathy Screening Instrument
NIHR	National Institute for Health Research
NPQ	Neuropathic Pain Questionnaire
NPSI	Neuropathic pain symptom inventory
NRSI	Nonrandomized studies of the effects of interventions
NF-kB	Nuclear factor kB
PPAR-α	Nuclear receptor peroxisome proliferator-activated receptor-α
NRS	Numeric rating scale
ODQ	Oswestry Disability Questionnaire
PEA	Palmitoylethanolamide
PICOS	Participants, intervention, control, outcome, study design
PRISMA	Preferred Reporting Items for Systematic Reviews and Meta-Analyses
RCTs	Randomized clinical trials
RoB	Risk of bias
ROBINS-I	Risk Of Bias In Non-randomised Studies of Interventions tool
TRPV1	Transient receptor potential channel of the vanilloid type 1
PEA-um	Ultramicronized PEA
VAS	Visual analogue scale
WOS	Web of Science
