# Supplementary material for: Effects of Palmitoylethanolamide (PEA) on Nociceptive, Musculoskeletal and Neuropathic Pain: Systematic Review and Meta-Analysis of Clinical Evidence
Source: Pharmaceutics. 2022 Aug 11;14(8):1672. doi: 10.3390/pharmaceutics14081672 (PMC9414729; doi:10.3390/pharmaceutics14081672)
Supplement: Supplementary file 1 [file pharmaceutics-14-01672-s001.zip › pharmaceutics-1790993-supplementary.pdf]

Graphical Overview for Evidence Reviews (GOfER) diagram of the systematic review and meta-analysis

DESIGN				PARTICIPANTS		EXPOSURE AND ASSESSMENT						STUDY QUALITY									STUDY FINDINGS: META-ANALYSIS
**Study report**	**Study design**	**Ethical approval**	**Sample size**	**Baseline characteristics**	**Inclusion criteria**	**Intervention**	**Control**	**Randomization, allocation and procedures to monitor or adherence/compliance**	**Primary outcome measure**	**Secondary outcome measure**	**Results**	**Randomization (RoB D1)**	**Baseline differences (ROBINS-I confounding and selection bias D1-D2)**	**Misclassification of intervention status (ROBINS-I D3)**	**Deviations from intended interventions (RoB D2 or ROBINS-I D4)**	**Missing outcome data (RoB D3 or ROBINS-I D5)**	**Bias in measurement of the outcome (RoB D4 or ROBINS-I D6)**	**Bias in selection of the reported results (RoB D5 or ROBINS-I D7)**	**Risk of bias assessment**	**Publication bias**	**Meta-analysis**
Andresen et al., 2016	Randomized, double blind, placebo-controlled, parallel multicenter trial NCT01851499	Approval by the Ethical Committee of the Central Denmark Region (no. 1-10-72-77-13) and the Danish Data Protection Agency, Copenhagen, Denmark (no. 1-16-02-106-13)	Based on sample size calculation, n=66. Total patients randomized n=73, but 5 patients did not meet the criteria of baseline pain intensity of 4 ≤ NRS ≤ 9, being excluded from primary intention-to-treat analysis	Average age 56.3 years (SD: 11.6); average time since injury 10.3 months (SD: 11.7); average pain intensity during baseline 6.4 (SD: 1.4) (NRS 0-10); patients treated with PEA-um used more rescue medications at baseline than control	Age≥18 years; traumatic or non-traumatic spinal cord injury (SCI, according to the International Standards for Neurological Classification of SCI) at least 6 months old; neuropathic pain (according to the the International Spinal Cord Injury Pain Basic Data Set) compatible with the SCI for at least 3 months with an average pain intensity of at	Sublingual ultramicronized PEA (PEA-um) 600 mg (Normast®), twice daily with approximately 12 hours between doses for 12 weeks, as add-on therapy. n=36	Identical placebo. n=37	Patients and investigators blinded; block computer-generated randomization with homogeneous block size of 4; patients returned the remaining sachets, recording data in case report form (CRF); completion of a pain diary	Difference in the mean value of the patient's daily ratings of average pain intensity in the baseline week and the last week of the treatment period, assessed with the NRS (0-10)	Average neuropathic pain intensity scores in the treatment weeks (weeks 2-12); the daily ratings of spasticity and sleep disturbance in the last week of the treatment period; muscle stiffness and spasms rated on a 0-10 NRS, use of rescue medication, neuropathic pain descriptors, impact of the neuropathic pain, healthrelated quality of life	No statistically significant difference in primary outcome (PEA 6.3±1.7 and 0.4±1.4 from baseline, Placebo 5.5±1.8 and 0.7±1.4 from baseline); significant reduction in the use of rescue medication; increased increase in self-reported intensity of spasticity; no statistically significant differences for all the other	+	——	——	+	+	+	+	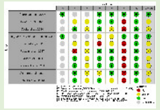	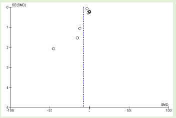	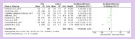

Graphical Overview for Evidence Reviews (GOfER) diagram of the systematic review and meta-analysis

					least 4 and not above 9 on a 0-10 point numeric rating scale (NRS)					using the Pain Survey (S-TOPS), evoked pain, spasticity assessed using the Modified Tardieu Scale (MTS), the Patient Global Impression of Change (PGIC), pain relief, the number needed to treat (NNT), the Insomnia Severity Index (ISI), the Major Depression Inventory (MDI) [2,3,27], and the Generalized Anxiety Disorder Assessment (GAD-10), adverse events assessed by open-ended	secondary outcomes; 5 patients reported serious adverse events; urinary tract infection, paralytic ileus, cholecystolithiasis, erysipelas causing hospitalization, fungus infection, blurred vision										

Graphical Overview for Evidence Reviews (GOfER) diagram of the systematic review and meta-analysis

										questions											
Bonetti et al., 2022	Observational	Written informed consent for paper publication.	____	Median age 47.2 years. Acute or chronic low-back pain/sciatica, unilateral with the innervation territories of L3 (19 patients), L4 (159 patients), L5 (105 patients), and S1 (35 patients). Patients with bilateral lower back and sciatic nerve pain or electromyographic features of neurogenic injury and/or denervation were excluded	Low back pain and/or sciatica and/or sciatic, with pain as by disc-radicular impingement from soft disc hernia (non-calcific)	Combined treatment of oxygen–ozone therapy and oral treatment with alpha-lipoic acid (ALA, 800mg/day) + palmitoylethanolamide (PEA, 600 mg/day) and myrrh (200 mg). n=153. three treatments with oxygen–ozone therapy over 30 days with distance of 9±2 days between the first and second therapeutic session and 18± 2 between the second and third.	Oxygen–ozone treatment alone. n=165. three treatments with oxygen–ozone therapy over 30 days with distance of 9 ±2 days between the first and second therapeutic session and 18 ±2 between the second and third.	____	Percentage of pain reduction	____	116/165 patients in Group A had a complete remission of pain (70.3%) while 21 (12.7%) and 28 (17.0%) had no benefit from the treatment, reporting a partial remission of painful symptoms, while in Group B 119/153 (77.8%) had a complete remission of pain, 13 (8.5%) considered the outcome of the treatment sufficient and 21 (13.7%) insufficient, 60 days 8 from the end of	____	-	-	+	+	X	+			

Graphical Overview for Evidence Reviews (GOfER) diagram of the systematic review and meta-analysis

											the treatment.										
Cocito et al., 2014	Open-label study	____	____	Visual Analogue Scale (VAS) mean score was 8.20 ± 1.53 (range 6–10), neuropathic pain symptom inventory (NPSI) total score was 5.2±1.5 (range 2. 4–8.7), and EQ-5D mean score was −0.30 ± 0.65 (range from −1.85 to 0.65).	Patients with diabetic or traumatic chronic neuropathic pain, not controlled by other oral conventional therapies, for at least 100 days at the time of recruitment, with a VAS pain score higher than 6, in spite of the best therapeutic regimen with Pregabalin, Gabapentin, and/or Tramadol. DN4 score ≥4, with an average score of 6.60 (range 4–9), and a stable intensity of pain in the preceding 100 days	Oral PEA-um treatment was initiated at the doses of 1200mg/die in sachet formulation for the first 10 days and 1200mg/die in tablet formulation between the 10th and 40th days. The dosages of all other therapies were maintained stable during the entire duration of the study. n=30	____	____	VAS evaluation was performed in all subjects after 10 days of treatment (T1), while VAS, NPSI, health questionnaire five dimensions (EQ-5D), and douleur neuropathique 4 (DN4) were repeated after 40 days of treatment	____	Significant decrease of the VAS mean score at the first evaluation (T1; 8.20 ± 1.53 vs 6.4 ± 1.83, $P < 0.002$), even more evident at the T2 evaluation (5.80 ± 2.04; $P < 0.001$). Significant improvement in the NPSI total score, from 5.2 ± 1.5 to the T2 (40 days) values of 3.8 ± 2.1 (P: 0.025), and similar trend for the EQ-5D mean score, from the T0 value of −0.30± 0.65 to T2 value of 0.50 ± 0.34	____	+	-	+	-	X	+			

Graphical Overview for Evidence Reviews (GOfER) diagram of the systematic review and meta-analysis

											(*P* < 0.001)										
Faig-Martì & Martinez-Catassus 2017	Prospective, double-blinded, randomized study	The study was previously approved by the Ethics Committee of the concerned Institution. Signed informed consent was required.	——	No statistical differences between the two groups in the baseline	Age between 18 and 75 years.Clinically and electrophysiologically confirmed diagnosis of low to moderate carpal tunnel syndrome for at least 3 months. Patients with a history of upper extremity surgery, currently taking steroids, using night splinting or with food allergies were excluded.	300 mg of PEA twice a day over 60 days. n=30	Placebo with exactly the same appearance twice a day for the same period. n=31	Yes	VAS pain assessment. Severity of symptoms and functional impairment of the patient assessed through a Spanish translation of Levine's questionnaire. Durkan's test, Phalen's test and electrophysiological data.	——	No significant differences in any outcomes. VAS 3.76 ± 3.19 (PEA) vs 3.25 ± 3.18 (Control)	+	——	——	-	-	+	+			
Gatti et al., 2012	Observational study	Approved by the Independent Ethics Committee of Fondazione Policlinico Tor Vergata	——	Caucasian with a mean age of 65.6 13.3 (432 females and 178 males). Therapies differed based	Patients resistant to chronic pain treatment (antidepressants, anticonvulsants, opioids and non-	PEA (600 mg) was administered twice daily for 3 weeks followed by single daily dosing	——	——	NRS pain intensity. Only in post-herpetic neuralgia patients, the NRS was assessed after	——	NRS significant decrease from 6.4 ± 1.4 to 2.5 ± 1.3. No treatment-related adverse events	——	-	-	X	-	X	+			

Graphical Overview for Evidence Reviews (GOfER) diagram of the systematic review and meta-analysis

		(Rome). All patients received a description of the study, prior to their giving written informed consent in accordance with the Declaration of Helsinki.		on patient's conditions. A minority of patients were not on a standard therapy	steroidal anti-inflammatory drugs) for chronic pain due to different pathological conditions for more than 6 months (with the exception of some patients affected by acute herpes zoster infection) with NRS ≥4 and age≥18, able to comprehend subjective pain scales	for 4 weeks, in addition to standard analgesic therapies or as single therapy. n=610			about 6 months from discontinuation of PEA treatment; Safety (discontinuation rates, treatment-emergent adverse events, and serious adverse events)		or serious adverse events										
Paladini et al., 2017	Observational study	Carried out in accordance with the Helsinki Declaration of 1964 and its subsequent revisions and Good Clinical Practice. All patients provided informed written	——	Patients selected for this study were already under treatment with tapentadol + pregabalin in the month before surgery, with a mean dosage of 150 mg and 300 mg,	Patients wth failed back surgery syndrome caused by laminectomy, discectomy, or vertebral stabilization, complaining of an increase in pain intensity compa	Tapentadol and pregabalin at variable doses, for three months in this study. One month after the start of standard treatment, um-PEA (Normast, Epitech	——	——	VAS evaluation every month for all patients at the time of enrollment ($T0$) and after one ($T1$), two ($T2$), and three ($T3$) months	——	VAS (2–8 months after surgery) 5.7 ± 0.12 vs VAS 4.3 ± 0.11 after 1 month of treatment (and 2.7 ± 0.09 after two and 1.7 ± 0.11 after 3 months of treatment) (for all measu	——	X	-	+	+	X	+	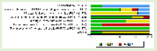		

Graphical Overview for Evidence Reviews (GOfER) diagram of the systematic review and meta-analysis

		consent to participate		respectively; the same dosages depending on the need of the patient were used in the prospective study. Therefore, there is confounding bias	red to the immediate postoperative condition	Group SpA, Saccolongo, Italia) was added at 1200mg/day (two 600mg tablets daily) for one month followed by 600 mg/day for the next month. n=35					res, *p* < 0.0001)										
Parisi et al., 2021	Prospective study	——	——	18 patients suffering from sciatic pain, 16 patients from carpal tunnel syndrome and 8 patients with peripheral neuropathy of the lower limbs were included in the Intervention group. 20 patients with sciatic pain, 15 with carpal tunnel syndrome and 5 with periph	Patients with rheumatic diseases with neuropathy from <12 months, documented by electromyography	Standard therapy + a fixed combination of PEA (600 mg) + Acetyl-L-Carnitine (500 mg) (Kalanit®) twice a day for 2 weeks and then once a day for 6 months. n=42	Standard therapy. n=40	——	Assessment after 3 months and after 6 months of treatment of: • VAS pain (0-10); •: Low Back Pain Impact Questionnaire (LBP-IQ; 0-100); •: cochin hand functional disability (CHFD; 0-90); •: Neuropathic Pain Questionnaire (NPQ; 1-12).	——	Significant improvement in pain VAS: intervention 5.8±1.3 vs 7.1±1.3 respect to standard therapy 6.1±0.7 vs 6.8±0.7. Significant improvement in LBP-IQ and CHFD scores.	——	+	-	+	+	X	+			

Graphical Overview for Evidence Reviews (GOfER) diagram of the systematic review and meta-analysis

				eral neuropathy of the lower limbsv, with same pathology and demographic characteristics respect to the Intervention group, were included in the control group																	
Passavanti et al., 2017	Pilot, observational study	The study was performed in compliance with the Good Clinical Practice guidelines and the principles of the Declaration of Helsinki of 1964 and its subsequent revisions; it followed STROBE guidelines for the reporting of observational studies	——	Not significant differences among groups for baseline characteristics, apart from chronic venous insufficiency of the lower limbs	Patients of age ≥ 18 years, with diagnosis of chronic low back pain, neuropathic pain for at least 6 months; stability of painful symptoms for at least 3 months; pain intensity score ≥ 6 measured by VAS, DN4≥4, hyperalgesia and allodynia by pinpric	Prospective arm: PEA-um as add-on therapy to tapentadol for 6 months. Paracetamol (1000 mg) was habitually used as rescue drug in case of exacerbations of pain. n=30	Retrospective arm: tapentadol for 6 months. Paracetamol (1000 mg) was habitually used as rescue drug in case of exacerbations of pain. n=25	——	Pain intensity (VAS), neuropathic component (DN4) at 3 weeks (T1), 12 weeks (T2) and 24 weeks; Degree of disability (Oswestry Disability Questionnaire, ODQ) at 3 weeks (T1), 12 weeks (T2) and 24 weeks	——	VAS significant reduction from 7.4 ± 0.08 to 4.5 ± 0.09 in the prospective group vs 7.7 ± 0.10 to 5.9 ± 0.09 in the retrospective group. DN4 mean score reduction from 6.1 ± 0.14 to 3.2 ± 0.13 with PEA vs from 6.1 ± 0.09 to 5.0 ± 0.04 in the retrospective group. Prospective	——	+	-	+	+	X	+			

Graphical Overview for Evidence Reviews (GOfER) diagram of the systematic review and meta-analysis

		and was communicated to the departmental Review Board.			k test and brush test						group presented ODQ reductionf from 56.9 ± 1.55 to 37.7 ± 2.38 vs retrospective group going from 54.6 ± 2.20 to 44.6 ± 3.02. PEA significantly reduced the dosage of tapentadol and the use of paracetamol. No serious side effects										
Scaturro et al., 2020	Observational Study	performed in accordance with the Good Clinical Practice guidelines and the principles of the Declaration of Helsinki of 1964 and its subsequent revisions. The study was approved by	——	37 males and 83 females) with a mean age of 56.4 ± 13.0 who were suffering from lumbosciatica and lumbocruralgia due to multiple herniated discs in the lumbar spine from 2 years	Patients aged ≥ 18 years old suffering from chronic pain due to multiple herniated discs in the lumbar spine with magnetic resonance confirm treated with analgesics (based general	PEA-um 600 mg twice a day in combination with a daily functional rehabilitation session +a decontracting massage for 20 consecutive days, followed by 600 mg of umPEA once a day for 40 days in	——	——	NRS pain intensity, 36-Item Short Form Health Survey questionnaire (SF-36) Questionnaire for quality of life and Oswestry Disability Questionnaire	——	NRS decreased significantly from 6.3 ± 0.1 at baseline to 3.7 ± 0.09 and 2 ± 0.09 at 30 and 60 days, respectively. Significant improvement in quality of life and mental component	——	+	-	-	+	X	+			

Graphical Overview for Evidence Reviews (GOfER) diagram of the systematic review and meta-analysis

		the Polyclinic University Hospital Paolo Giaccone Ethics Committee (report number 05/2019). Written consent for participation was obtained from each patient after they had been fully informed of the purpose of the study			ly on gabapentinoids or opioids), albeit with poor results and maintained during the whole study	addition to standard therapy. n=120															
Schifilliti et al., 2014	Open-label study	Performed in accordance with guidelines established for Good Clinical Practice, the study protocol was communicated to the health care managers of the MOV.I.S.	——	Mean age of 68.3 ± 9.4 years) affected by Type II diabetes (mean time from onset 18.2 ± 9.0 years) and complaining of neuropathic painful symptoms	Diabetic patients suffering from painful diabetic neuropathy	Micronized palmitoylethanolamide (300mg twice daily) for 60 days. n=30	——	——	Michigan Neuropathy Screening instrument (MNSI); Total Symptom Score (TSS) for diabetic neuropathic pan symptoms; Neuropathic Pain Symptoms Invent	——	Significant reduction in the pain symptoms characteristic of diabetic neuropathy already after 30 days (MNSI, TSS, NPSI). No serious adverse events	——	+	-	+	-	X	+			

Graphical Overview for Evidence Reviews (GOfER) diagram of the systematic review and meta-analysis

		Onlus. The study was conducted, according to the ethical principles set out in the Declaration of Helsinki and its revisions. All eligible patients were properly informed about the study and gave their written informed consent to participate							ory (NPSI); Hematological and blood tests to evaluate metabolic control and safety chemistry												
